# Dietary Intakes of Animal and Plant Proteins and Risk of Colorectal Cancer: The EPIC-Italy Cohort

**DOI:** 10.3390/cancers14122917

**Published:** 2022-06-13

**Authors:** Sabina Sieri, Claudia Agnoli, Valeria Pala, Sara Grioni, Domenico Palli, Benedetta Bendinelli, Alessandra Macciotta, Fulvio Ricceri, Salvatore Panico, Maria Santucci De Magistris, Rosario Tumino, Luigi Fontana, Vittorio Krogh

**Affiliations:** 1 Epidemiology and Prevention Unit, Fondazione IRCCS Istituto Nazionale dei Tumori, 20133 Milan, Italy; claudia.agnoli@istitutotumori.mi.it (C.A.); valeria.pala@istitutotumori.mi.it (V.P.); sara.grioni@istitutotumori.mi.it (S.G.); vittorio.krogh@istitutotumori.mi.it (V.K.); 2 Institute for Cancer Research, Prevention and Clinical Network (ISPRO), 50139 Florence, Italy; d.palli@ispro.toscana.it (D.P.); b.bendinelli@ispro.toscana.it (B.B.); 3 Centre for Biostatistics, Epidemiology and Public Health (C-BEPH), Department of Clinical and Biological Sciences, University of Turin, 10124 Turin, Italy; alessandra.macciotta@unito.it (A.M.); fulvio.ricceri@unito.it (F.R.); 4 Dipartimento Di Medicina Clinica E Chirurgia, Federico II University, 80138 Naples, Italy; spanico@unina.it; 5 Dipartimento Assistenziale Integrale di Medicina Interna ad Indirizzo Specialistico, Azienda Ospedale Universitaria Federico II, 80131 Naples, Italy; masantuc@unina.it; 6 Hyblean Association for Epidemiological Research, AIRE ONLUS, 97100 Ragusa, Italy; rosario.tumino@asp.rg.it; 7 Charles Perkins Centre, Faculty of Medicine and Health, University of Sydney, Sydney, NSW 2006, Australia; luigi.fontana@sydney.edu.au; 8 Department of Endocrinology, Royal Prince Alfred Hospital, Sydney, NSW 2006, Australia; 9 Department of Clinical and Experimental Sciences, Brescia University School of Medicine, 25121 Brescia, Italy

**Keywords:** protein intake, animal protein, vegetable protein, glycemic index, colorectal cancer

## Abstract

**Simple Summary:**

After breast and prostate cancer, colorectal (CRC) is the third most frequent cancer in men and women. It is unclear if protein-rich diets other than red meat elevate risk or even lower CRC occurrence at specific colon locations. The aim of this study is to assess the associations of animal and plant protein intakes with CRC risk in middle-aged Italian men and women. Our findings show that replacing animal proteins with plant proteins was associated with a lower risk of rectal cancer but not of colon cancer, while replacing animal proteins with plant-based proteins from high-glycemic-index (GI) foods was associated with an increased colon cancer risk. These results have important public health implications as they suggest that both refined high-GI foods and meat might have site-specific roles in the pathogenesis of CRC.

**Abstract:**

We prospectively investigated the associations of protein intake with colorectal cancer (CRC) risk in middle-aged Italian men and women. Food consumption was assessed by validated Epic semiquantitative food-frequency questionnaires. Multivariable Cox models stratified by center, age, and sex, adjusted for confounders, estimated the associations of animal and plant protein consumption with CRC risk by subsite. Among 44,824 men and women, we identified 539 incident CRCs after a median follow-up of 14 years. Replacing animal proteins with plant proteins was associated with a decreased risk of rectal (HR, 0.71; 95% CI, 0.55–0.92) but not colon cancer. By contrast, replacing animal proteins with plant proteins from high-glycemic-index (GI) foods was associated with an increased risk of proximal and distal (including sigma) colon cancer (HR, 1.23; 95% CI, 1.07–1.40) but not when animal proteins were replaced with plant proteins from low-GI foods (HR, 0.93; 95% CI, 0.79–1.11). Further evaluation revealed that the increased colon cancer risk was limited to the substitution of proteins from red and processed meat, as well as dairy and eggs, with vegetable proteins from high-GI foods. Participants in the highest quintile of animal protein intake had higher plasma glucose and cholesterol levels than those in the lowest quintile. By contrast, higher intake of plant proteins from low-GI foods was inversely associated with fasting insulin and HOMA-IR levels. In conclusion, replacing animal proteins with plant proteins from high-GI foods was associated with an increased risk of colon cancer.

## 1. Introduction

Colorectal cancer (CRC) is the third most frequent malignant tumor in both men and women, and its incidence rates are almost nine-fold higher in high-income countries than in developing countries [1]. It has been hypothesized that modifiable risk factors, especially unhealthy diets, are responsible for most of this striking variation in rates and for the recent rise in the incidence of early-onset CRC [2].

Data from case-control and cohort studies strongly suggest that Western dietary patterns characterized by a higher consumption of red and processed meats and a lower intake of fiber-rich foods such as vegetables and fruits are associated with an increased CRC risk, especially in young age groups [3,4]. However, it is unclear if protein-rich diets other than red meat elevate risk or might even lower CRC occurrence at specific colon locations.

Fecal content and gut microbiota composition gradually change from the cecum to the rectum as water is absorbed, and colon and rectum mucosa exposure to dietary carcinogens [5] and bacterial fermentation byproducts increases [6]. These changing interactions with mucosal epithelial and immune cells may directly cause genetic and epigenetic molecular alterations that can have a major influence on tumor initiation and progression at bowel subsites [7].

In this analysis, we used data from a large Italian cohort study (EPIC-Italy, 1993–1998) of 44,824 men and women, with up to 14 years of follow-up, to prospectively examine the associations of animal and plant protein intakes with the risk of CRC overall and by anatomic subsites. We also performed an assessment, using statistical methods, of the effect of the isocaloric replacement of proteins from one source with proteins from another source on CRC incidence and analyzed the joint association of protein intake and lifestyle factors (smoking, body mass index, physical activity, alcohol consumption) with location-specific incidence rates.

## 2. Materials and Methods

### 2.1. Study Population

EPIC-Italy is the Italian section of the European Prospective Investigation into Cancer and Nutrition, an ongoing multi-center prospective cohort study to investigate the role of diet on the development of cancer in 10 European countries [8]. A total of 47,749 volunteers were recruited to take part in EPIC-Italy mainly between 1993 and 1998 in five centers: two in northern Italy (Varese and Turin), one in central Italy (Florence), and two in southern Italy (Ragusa and Naples), as described elsewhere [9].

Diet (semi-quantitative food-frequency questionnaires), lifestyle, anthropometric and clinical measurements, and blood samples were obtained from each participant after they signed an informed consent form. Two thousand nine hundred twenty-five participants were lost track of for follow-up. Prevalent cancer cases with no dietary or lifestyle information and/or anthropometric data, or with extreme values of the ratio of total energy intake to basal metabolic rate (cut-offs of the first and last half-percentiles) were excluded, leaving a total of 44,824 participants for analysis.

### 2.2. Follow-Up

The EPIC-Italy database was linked to the cancer and regional mortality registries after appropriate database quality checks. All EPIC-Italy centers except for Naples are covered by population-based cancer registries. In Naples, linkage to electronic hospital discharge records and periodic personal contact with participants were carried out to collect follow-up information.

Participants were followed-up from the date of entry in the cohort until the occurrence of any cancer (except non-melanoma skin cancer), death, emigration, or end of follow-up, whichever occurred first. Follow-up ended on 31 December 2009, in Varese; on 31 December 2010, in Florence and Naples; and on 31 December 2014, in Ragusa and Turin. The final study follow-up date varied for each center due to availability of up-to-date cancer registry and hospital discharge files.

Colon cancers were identified by the codes of the International Classification of Diseases (10th Revision [10]) as follows: proximal (C18.0–18.5), distal (C18.6–C18.7), overlapping (C18.8), and unspecified (C18.9) sites. Rectal cancers were identified by codes C19 (rectosigmoid junction) and C20 (rectum). Anal cancers were excluded.

### 2.3. Dietary Assessment

Validated center-specific food-frequency questionnaires (FFQs) were administered to capture detailed information about all foods and beverages consumed by participants in the year before recruitment, as described elsewhere [11]. The nutrient contents of all food items included in the FFQs were obtained from Italian food composition tables [12].

Intakes of animal and vegetable proteins were expressed as percentages of the total energy intake. Processed and unprocessed red meat (36%), poultry (13.1%), dairy products (34.1%), fish (10.9%), and eggs (3.9%) were the main sources of animal proteins, whereas bread (42.7%), pasta (19.3%), pizza (3.4%), rice (2.6%), vegetables (9.7%), fruits (7.3%), legumes (5%), and potatoes (2%) were the main sources of vegetable proteins.

Vegetable protein intake was further divided according to whether it came from foods with high or low glycemic index (GI), using a GI of 55 as the cut-off value [13]. Bread, pizza, rice, and potatoes were the main sources of vegetable proteins from high-GI foods; pasta, vegetables, fruits and legumes were the main sources of vegetable proteins from low-GI foods.

The contribution of any one food category to the intake of animal or vegetable proteins was calculated for each individual participant as the percentage of animal or vegetable protein intake relative to the total animal or vegetable protein intake. The vegetable protein contents of high- and low-GI foods were calculated by multiplying the protein contents of each food by the average quantity of that food consumed daily.

### 2.4. Other Study Variables

Anthropometric variables such as weight (kg), height (m), and BMI (kg/m^2^) were measured at enrolment according to the EPIC protocol. Physical activity was categorized according to the Cambridge Physical Activity Index [14].

A comprehensive medical history including medication use, hormonal treatment, menopausal status, physical activity, alcohol consumption, smoking, and education was collected through a standardized lifestyle questionnaire administered at recruitment.

In a subset of 2402 participants randomly selected from four Italian EPIC centers (Varese, Turin, Naples, and Ragusa), the plasma concentrations of fasting glucose, triglycerides, and lipoprotein-cholesterol were determined with automated enzymatic colorimetric commercial kits (Instrumentation Laboratory); fasting insulin was measured using ELISA kits (DRG Instruments GmbH, Germany), and high-sensitivity C-reactive protein was measured using a latex particle-enhanced immunoturbidimetric assay (IL Coagulation Systems on ACL9000). HOMA-IR was calculated as fasting glucose divided by fasting insulin [15].

### 2.5. Statistical Methods

The distributions of the participants’ characteristics are presented as means and standard deviations (continuous variables) or percentages (categorical variables) by quintiles of energy-adjusted intakes of proteins from animals, from high-GI vegetables, and from low-GI vegetables.

Hazard ratios (HRs) with 95% confidence intervals (CIs) for colon and rectum cancers, in relation to the intakes of total animal proteins and animal proteins from different sources, were estimated by Cox regression modeling, with age as the underlying time variable, stratified by center (Varese, Turin, Florence, Naples, and Ragusa) to control for center effects and by age at recruitment (1-year categories) to account for possible departures from the proportionality of hazards with time and sex. Entry time was age at recruitment and exit time was age at first colorectal event, death, or censoring date (loss or end of follow-up), whichever occurred first. Animal and vegetable protein intakes were adjusted for energy intake and percentage of fats using the nutrient density method [16].

The effects (on cancer risk) of replacing 3% of energy from animal proteins with equivalent amounts from vegetable proteins, vegetable proteins from high-GI foods, or vegetable proteins from low-GI foods were then estimated by simultaneously including the animal-protein-derived energy and the energy from one of the plant protein items as continuous variables in a multivariable model. We then calculated the difference between the two coefficients, accounting for their variance and covariance, and exponentiated the difference to afford the HR and 95% CI for the replacement [17].

Potential confounders were chosen based on prior knowledge. We ran minimally adjusted models including non-alcoholic energy intake and alcohol consumption (<12, 12–24, >24 g/day) as covariates and adjusted models that also included the following covariates: smoking status (current: 1–15 cig/day, 16–25 cig/day, 26+ cig/day; former: quit ≤10 years, 11–20 years, 20+ years previously, never), physical activity (inactive, moderately inactive, moderately active, active), body mass index (BMI; kg/m^2^), waist-to-hip ratio, years of education (<8 years/≥8 years), and fiber intake (g/day).

The proportional hazards assumption for all variables in relation to colon and rectal cancer risk was tested using the Grambsch and Therneau method [18]. In all cases, the assumption was satisfied.

We also examined whether associations between animal proteins, and colon and rectal cancers were modified by the BMI. This was achieved by modeling the product terms of the dichotomized BMI variable (≤25 and >25) multiplied by the participant’s animal/animal food source intake considered as a continuous variable (Supplementary Tables S1 and S2). The significance of the interaction was assessed by comparing the likelihood ratio test statistic of the models, with and without the product term, to a chi-squared distribution with one degree of freedom. All analyses were conducted using Stata software (version 16.0; Stata Corp, College Station, TX, USA).

## 3. Results

After a median follow-up of 14 years, 539 cohort participants were diagnosed with colorectal cancer (438 colon and 101 rectum). Table 1 shows the baseline characteristics of study participants by the quintiles of the energy-adjusted intakes of animal proteins and vegetable proteins from low- and high-GI foods. Mean energy from animal proteins varied from 6.4% in the lowest to 14% in the highest quintile, whereas mean energy from vegetable proteins from high- and low-GI foods ranged approximately from 4% to 7%.

Participants in the highest quintiles of animal proteins consumed more fats and less starch, sugar, fiber, alcohol, and energy; had higher BMI and lower waist-to-hip ratio; and were less educated and physically active than those in lower quintiles. Participants in the highest quintile of energy from animal proteins consumed more proteins from processed and unprocessed red meat, poultry, fish, egg, and dairy but less fruits, pasta, bread, and pizza.

Participants in the highest quintiles of dietary intakes of vegetable proteins from high- and low-GI foods consumed more starch and fiber and less alcohol, whereas energy intake did not vary greatly across quintiles. Participants in the highest quintile of dietary vegetable proteins from high-GI foods consumed less fats. The BMI and waist-to-hip ratio were higher in participants with a higher intake of vegetable proteins from high- and low-GI foods. Participants in the highest quintile of energy from vegetable proteins from high-GI foods consumed more proteins from bread and pizza but less from red and processed meat, poultry, fish, egg, and dairy, whereas participants in the highest quintile of energy from vegetable proteins from low-GI foods consumed more proteins from pasta, vegetable, legumes, fruits, and fish.

Participants in the highest quintile of intake of energy from animal proteins had significantly higher fasting glucose and cholesterol than participants in the lowest quintile. In contrast, those in the highest quintile of energy from vegetable proteins from low-GI foods but not from high-GI foods had lower insulin and HOMA-IR than participants in the lowest quintile (Table 1).

As shown in Table 2, no associations were observed between the replacement of animal proteins with vegetable proteins and colon cancer risk. When vegetable proteins were split into vegetable proteins from high- and low-GI foods, only the replacement of 3% of energy from animal proteins with vegetable proteins from high-GI foods, and not with energy from vegetable proteins from low-GI foods, was associated with an increased colon cancer risk. After adjusting for major lifestyle and dietary risk factors, the HR for the replacement of animal proteins with vegetable proteins from high-GI foods was 1.23 (CI 95% = 1.07–1.40).

By contrast, the replacement of 3% of energy from animal proteins with vegetable proteins was associated with a decreased rectal cancer risk (HR, 0.71; 95% CI = 0.55–0.92; adjusted model). The protective effect was confined to the replacement of animal proteins with vegetable proteins from high-GI foods (HR, 0.68; 95% CI = 0.51–0.89; adjusted model).

Table 3 shows the risk of colon and rectal cancers associated with the replacement of 3% of energy from different animal protein sources with plant proteins.

The HR (95% CI) for colon cancer was 1.21 (95% CI = 1.07–1.39) when 3% of energy from proteins from processed red meat was substituted with an equivalent amount of vegetable proteins. The substitution with vegetable proteins from high- but not low-GI foods showed stronger associations with colon cancer. The substitution of 3%-energy proteins from processed and red meat, and dairy and eggs with an equivalent amount of vegetable proteins from high-GI foods was associated with 32% (95% CI = 1.14–1.52) and 22% (95% CI = 1.06–1.40) increases in the risk of colon cancer, respectively. No associations were found when vegetable proteins replaced fish. By contrast, the substitution of proteins from processed and red meat, poultry, and eggs plus dairy with an equivalent amount of vegetable proteins from high-GI foods only was associated with a decreased risk of rectal cancer (Processed and red meat: HR, 0.66; 95% CI = 0.50–0.87. Poultry: HR, 0.70; 95% CI = 0.50–0.96. Eggs and dairy: HR, 0.74; 95% CI = 0.57–0.97.). There were no protective effects following the replacement with vegetable proteins from high-GI foods.

Finally, we tested whether the association of colon and rectal cancers with proteins was modified by BMI status. We found no influences of the BMI on the relation between animal proteins (Table S1) or animal protein food sources (Table S2) and colon cancer (tests for interaction: *p* = 0.826 for animal proteins replaced with vegetable proteins and *p* = 0.754 for animal proteins replaced with vegetable proteins from high- and low-GI foods) or rectal cancer (tests for interaction: *p* = 0.797 for animal proteins replaced with vegetable proteins and *p* = 0.820 for animal proteins replaced with vegetable proteins from high- and low-GI foods) risk. However, an increased risk of colon cancer for the replacement of animal proteins with vegetable proteins and a reduced risk of rectal cancer were confined to participants with a BMI ≤ 25.

## 4. Discussion

In this large cohort study of 44,824 Italian men and women followed for a mean of 14 years, we found that replacing animal proteins with plant proteins was associated with an approximately 30% lower risk of rectal but not colon cancer. Substituting animal proteins with plant proteins from high- but not low-GI foods was associated with a 23% higher risk of colon cancer.

A higher intake of vegetable proteins from low-GI foods was associated with lower plasma insulin levels and insulin resistance. By contrast, high animal protein intake was associated with elevated plasma cholesterol and glucose levels, even though these participants consumed significantly less calories and had a BMI similar to those with low animal protein intake, suggesting that the protein source plays a crucial role in modulating metabolic health and CRC risk, irrespective of body weight.

It remains controversial whether animal proteins promote colorectal cancer, although the International Agency for Cancer Research has classified red meat as a probable human carcinogen and processed meat as a known human carcinogen [19,20]. Some epidemiological studies have suggested a positive association between red meat intake and CRC, but other studies found no associations, possibly because of the high heterogeneity in dietary patterns and primary tumor location [21,22,23]. Our findings suggest that the substitution of energy from animal proteins, including processed and unprocessed red meat, poultry, eggs, and dairy (but not fish), with an equivalent amount of vegetable proteins was associated with an increased risk of rectal cancer but not of colon cancer in a cohort of Italian men and women that consumed substantially less animal proteins than people from North America.

In the ~150 cm long large intestine, water gets progressively absorbed while the resident microbiotas complete the process of the chemical digestion of proteins and other nutrients, and the intestinal content is pushed towards the 16–18 cm long anorectal canal. Previous studies have shown that compared with the proximal colon, DNA and alkylating damage to key genes such as KRAS and PI3K from exposure to dietary carcinogens is higher in both normal crypts and tumors of the distal colon, probably as a result of long-term feces storage and contact with the columnar and stratified squamous epithelium in this terminal portion of the large intestine [7]. Consistently, in our study, we found an increased risk of rectal tumors in men and women with a higher intake of animal proteins. By contrast, the substitution of animal proteins with plant proteins from high-GI, low-fiber foods was associated with a higher cancer risk at all the other colon subsites, possibly through insulin resistance and the compensatory hyperinsulinemia-mediated stimulation of the PI3K/AKT/AMPK pathway [24,25,26]. The increased risk of colon cancer we found when animal proteins were replaced by vegetable proteins from high-GI foods is consistent with our finding in a previous analysis of the same Italian EPIC cohort. i.e., the high consumption of carbohydrates from high-GI foods was associated with an increased risk of cancer at all colon sites but not rectum [27]. Together, these data suggest that GI may be a major determinant of colon cancer risk in our cohort, characterized by the consumption of a large variety of plant proteins from low- and high-GI foods.

A detrimental synergistic interaction of different protein sources and their related food constituents with an individual’s specific gut microbiome might explain at least in part the inconsistent results across sites and protein sources. Total dietary protein intake and insoluble fiber profoundly shape gut bacterial species abundances and their function in both animals and humans [28], which, in turn, might alter host metabolic and physiologic responses to other food constituents and the energy intake itself [29,30,31]. Recent studies have shown a causal relationship between a prolonged exposure of host epithelial cells to colibactin from genotoxic E. coli strains and mutational signatures in primary human colon crypts [32], which suggests that functional features in host intestinal environments (e.g., molecular characteristics of mucosal surfaces) can be influenced by specific bacterial taxa and microbiome functions.

The present study has several strengths and limitations. Our analysis was performed on a large sample of Italian men and women who were followed for 14 years and ate substantially less animal proteins than cohorts from North America [33]. The availability of a range of lifestyle factors allowed us to perform confounding adjustment and subgroup analyses by colon subsites. Metabolic, hormonal, and inflammatory biomarkers provided a distinctive mechanistic insight into these associations. The calculation of protein intake according to food source and the analyses of the effect of substituting proteins of various origins are other strengths of our study. The observational nature of our study with potential measurement errors in dietary assessment and residual or unmeasured confounding are limitations that were minimized by the multivariable adjustment and stratified analyses according to lifestyle. Another limitation is that diet was only assessed at baseline. A further limitation is that we could not examine the relations among food mutagens, gut microbiome composition, and CRC risk.

## 5. Conclusions

In summary, our data suggest that replacing animal proteins with plant proteins is associated with a reduced risk of rectal cancer. In the colon, however, plant proteins from high-GI foods seem to be responsible for a higher risk of cancer. These findings have important public health implications because they suggest that both refined and processed high-GI foods and meat have a site-specific role in the pathogenesis of colorectal cancer. More studies are warranted to elucidate the interactive roles of dietary changes in metabolism and in gut microbiome structure and function in CRC biology.

## Figures and Tables

**Table 1:** Mean (SD) or frequencies (%) of selected variables according to percentage of energy from animal proteins, vegetable proteins from high-GI foods, and vegetable proteins from low-GI foods.

	Animal Proteins				Vegetable Proteins from High-GI Foods				Vegetable Proteins from Low-GI Foods			
	<8%	>9, <11%	>12%		<2%	>2.5, <3.1%	>3.8%		<1.5%	>1.9; <2.2%	>2.8%	
	Quintile 1	Quintile 3	Quintile 5	*p*-Value ^§^	Quintile 1	Quintile 3	Quintile 5	*p*-Value ^§^	Quintile 1	Quintile 3	Quintile 5	*p*-Value ^§^
Characteristics												
Participants (n)	9000	8999	8999		9000	8999	8999		9000	8999	8999	
Age	49.8 (8.12)	50.5 (7.91)	51.5 (7.66)	<0.001	50.6 (7.9)	50.5 (7.9)	50.6 (7.9)	0.469	50.7 (8.0)	50.4 (7.9)	50.7 (7.8)	0.048
Gender												
Male (%)	39.3	31.6	22.0		26.5	31.5	36.6		28.8	34.0	27.2	
Female (%)	60.7	68.4	78.0	<0.001	73.5	68.5	63.4	<0.001	71.2	66.0	72.8	<0.001
Center												
Turin (%)	16.9	21.6	24.8		24.7	23.4	13.3		22.2	23.3	17.2	
Varese (%)	18.0	27.6	31.9		37.5	28.2	9.6		32.9	27.0	15.6	
Florence (%)	24.3	28.9	32.7		26.1	30.4	27.9		28.9	31.3	20.7	
Naples (%)	10.2	12.6	7.3		8.4	10.0	14.2		0.38	3.1	40.3	
Ragusa (%)	30.6	9.3	3.3	<0.001	3.2	7.8	35.1	<0.001	15.6	15.2	6.1	<0.001
BMI (kg/m^2^)	25.6 (3.94)	25.9 (4.00)	26.6 (4.23)	<0.001	25.8 (4.0)	25.9 (4.0)	26.3 (4.2)	<0.001	25.8 (4.1)	25.9 (4.0)	26.3 (4.2)	<0.001
Waist-to-hip ratio	0.85 (0.09)	0.83 (0.09)	0.83 (0.09)	<0.001	0.82 (0.09)	0.83 (0.09)	0.86 (0.09)	<0.001	0.83 (0.09)	0.84 (0.09)	0.84 (0.08)	<0.001
Current smoker (%)	21.6	19.5	18.5	<0.001	20.3	19.5	20.9	<0.001	20.6	19.1	22.3	<0.001
Physical activity												
Inactive (%)	29.6	29.0	28.8		25.4	27.8	35.9		24.3	24.4	45.9	
Moderately inactive (%)	34.5	38.6	42.1		40.4	39.0	33.9		42.6	40.2	27.7	
Moderately active (%)	17.9	18.0	16.6		18.5	18.6	15.6		18.5	19.1	13.7	
Active (%)	18.0	14.4	12.5	<0.001	15.7	14.6	14.2	<0.001	14.5	16.3	16.9	<0.001
Education (>8 years)	20.4	20.4	18.7	<0.001	19.5	21.1	18.8	<0.001	20.7	19.6	20.3	<0.001
Diastolic Pressure (mmHg)	80.6 (9.8)	81.9 (10.0)	82.6 (10.3)	<0.001	81.9 (10.0)	82.0 (10.2)	81.0 (9.98)	<0.001	81.8 (10.1)	81.7 (10.1)	81.9 (10.0)	<0.001
Systolic Pressure (mmHg)	127.7 (17.7)	129.6 (18.1)	130.7 (18.5)	<0.001	129.5 (17.9)	129.8 (18.2)	128.7 (18.1)	<0.001	128.7 (17.6)	128.9 (17.9)	131.6 (19.3)	<0.001
Dietary intake												
Total proteins (% E/d) ^#^	14.0 (1.35)	16.6 (1.00)	20.0 (1.74)	<0.001	17.1 (2.7)	16.9 (2.3)	16.1 (2.0)	<0.001	16.8 (2.5)	16.8 (2.4)	16.7 (2.3)	<0.001
Animal proteins (%E/d) ^#^	6.41 (1.16)	9.97 (0.40)	14.3 (1.80)	<0.001	11.4 (3.1)	10.4 (2.6)	8.3 (2.3)	<0.001	10.6 (3.1)	10.3 (2.8)	9.4 (2.6)	<0.001
Vegetable proteins (%E/d) ^#^	6.00 (1.44)	5.10 (1.12)	4.36 (0.97)	<0.001	3.9 (0.9)	5.0 (0.8)	6.7 (1.0)	<0.001	4.5 (1.3)	5.0 (1.1)	6.0 (1.1)	<0.001
Total fats (% E/d) ^#^	30.4 (5.80)	34.1 (4.84)	37.9 (5.17)	<0.001	38.6 (5.3)	34.4 (4.6)	29.1 (4.5)	<0.001	33.7 (5.8)	34.4 (5.6)	33.9 (5.6)	<0.001
Starch (% E/d) ^#^	33.0 (7.77)	27.5 (6.19)	22.0 (5.88)	<0.001	20.1 (5.5)	26.9 (4.6)	36.5 (5.6)	<0.001	26.6 (7.7)	27.0 (7.2)	29.8 (7.3)	<0.001
Sugar (% E/d) ^#^	18.3 (6.05)	17.6 (5.25)	16.7 (4.98)	<0.001	20.1 (6.0)	17.5 (5.0)	15.1 (4.5)	<0.001	18.0 (5.8)	17.7 (5.3)	16.7 (5.1)	<0.001
Alcohol (% E/d) ^#^	4.3 (5.4)	4.2 (5.1)	3.3 (4.5)	<0.001	4.1 (5.4)	4.3 (5.1)	3.2 (4.3)	<0.001	4.9 (5.9)	4.1 (4.9)	2.9 (4.0)	<0.001
Fiber (g/day)	25.9 (8.8)	22.3 (6.9)	18.33 (6.0)	<0.001	20.2 (7.4)	21.9 (7.2)	24.9 (8.0)	<0.001	19.1 (6.7)	22.3 (7.2)	25.6 (8.2)	<0.001
Total energy intake (kcal/day)	2451 (696)	2341 (640)	2058 (610)	<0.001	2234 (676)	2299 (655)	2374 (669)	<0.001	2313 (701)	2309 (665)	2299 (606)	0.013
Protein Sources												
Red Meat (% E/d) ^#^	1.4 (0.9)	2.5 (1.2)	3.9 (1.9)	<0.001	2.9 (1.8)	2.6 (1.5)	2.1 (1.3)	<0.001	2.7 (1.7)	2.6 (1.6)	2.3 (1.4)	<0.001
Processed meat (% E/d) ^#^	0.6 (0.5)	1.0 (0.7)	1.3 (1.0)	<0.001	1.1 (0.8)	1.0 (0.7)	0.8 (0.7)	<0.001	1.2 (0.9)	1.0 (0.7)	0.7 (0.6)	<0.001
Poultry (% E/d) ^#^	0.8 (0.6)	1.3 (0.8)	2.1 (1.4)	<0.001	1.5 (1.2)	1.4 (1.1)	1.2 (0.9)	<0.001	1.3 (1.0)	1.5 (1.1)	1.4 (1.0)	<0.001
Fish (% E/d) ^#^	0.7 (0.6)	1.1 (0.7)	1.6 (1.2)	<0.001	1.3 (1.0)	1.2 (0.9)	1.0 (0.8)	<0.001	1.0 (0.8)	1.1 (0.9)	1.4 (1.0)	<0.001
Eggs (% E/d) ^#^	0.3 (0.2)	0.4 (0.2)	0.5 (0.3)	<0.001	0.4 (0.3)	0.4 (0.3)	0.3 (0.2)	<0.001	0.4 (0.3)	0.4 (0.30)	0.4 (0.2)	<0.001
Dairy (% E/d^#^)	2.4 (1.1)	3.6 (1.3)	4.8 (1.9)	<0.001	4.2 (1.8)	3.7 (1.6)	2.8 (1.3)	<0.001	4.0 (1.9)	3.6 (1.6)	3.2 (1.4)	<0.001
Potatoes (% E/d) ^#^	0.10 (0.09)	0.10 (0.08)	0.10 (0.08)	<0.001	0.11 (0.09)	0.10 (0.08)	0.10 (0.07)	<0.001	0.08 (0.06)	0.11 (0.08)	0.12 (0.09)	<0.001
Vegetables (% E/d) ^#^	0.59 (0.36)	0.62 (0.32)	0.68 (0.34)	<0.001	0.73 (0.39)	0.63 (0.32)	0.53 (0.27)	<0.001	0.42 (0.18)	0.62 (0.28)	0.86 (0.43)	<0.001
Legumes (% E/d) ^#^	0.16 (0.32)	0.19 (0.35)	0.15 (0.26)	<0.001	0.18 (0.33)	0.17 (0.31)	0.17 (0.31)	<0.001	0.05 (0.06)	0.10 (0.11)	0.51 (0.56)	<0.001
Fruits (% E/d) ^#^	0.44 (0.28)	0.39 (0.21)	0.37 (0.20)	<0.001	0.43 (0.26)	0.39 (0.22)	0.37 (0.22)	<0.001	0.30 (0.15)	0.41 (0.22)	0.47 (0.28)	<0.001
Pasta (% E/d) ^#^	0.96 (0.67)	0.87 (0.57)	0.66 (0.51)	<0.001	0.89 (0.68)	0.86 (0.58)	0.76 (0.49)	<0.001	0.35 (0.23)	0.79 (0.37)	1.42 (0.72)	<0.001
Rice (% E/d) ^#^	0.13 (0.18)	0.15 (0.16)	0.14 (0.16)	<0.001	0.13 (0.13)	0.16 (0.17)	0.12 (0.17)	<0.001	0.13 (0.17)	0.15 (0.17)	0.14 (0.14)	<0.001
Bread (% E/d) ^#^	3.00 (1.49)	2.14 (1.06)	1.58 (0.90)	<0.001	0.83 (0.45)	2.03 (0.44)	4.01 (0.97)	<0.001	2.47 (1.41)	2.22 (1.18)	2.00 (1.10)	<0.001
Pizza (% E/d) ^#^	0.20 (0.18)	0.18 (0.15)	0.16 (0.14)	<0.001	0.16 (0.13)	0.18 (0.14)	0.21 (0.19)	<0.001	0.18 (0.16)	0.18 (0.15)	0.18 (0.14)	<0.001
Plasma Biomarkers *												
Participants (n)	540	504	520		553	493	515		529	508	517	
Insulin (mU/L)	9.62 (5.93)	9.79 (7.92)	9.69 (6.28)	0.543	10.2 (9.4)	9.53 (6.72)	10.0 (7.76)	0.237	10.7 (8.80)	9.7 (5.64)	8.25 (7.27)	<0.001
Blood glucose (mg/dl)	97.9 (27.7)	100.1 (31.1)	104.3 (39.4)	0.002	100.2 (26.8)	99.4 (31.0)	101.1 (29.2)	0.554	100.6 (24.6)	100.2 (25.9)	98.3 (31.7)	0.540
HOMA-IR	2.02 (1.54)	2.18 (2.70)	2.21 (1.98)	0.512	2.26 (3.43)	2.10 (2.39)	2.22 (2.40)	0.258	2.37 (2.63)	2.10 (1.62)	1.83 (2.14)	0.004
Cholesterol (mg/dl)	227.8 (46.0)	234.3 (47.4)	241.3 (47.7)	<0.001	241.8 (48.6)	234.5 (50.1)	231.8 (48.8)	<0.001	235.3 (45.5)	237.3 (45.5)	231.6 (50.7)	0.324
Triglycerides (mg/dl)	146.8 (81.8)	138.2 (79.8)	143.9 (97.3)	0.529	141.7 (96.2)	146.0 (91.9)	150.7 (84.3)	0.187	144.9 (97.0)	141.2 (78.7)	144.9 (94.7)	0.934
C reactive protein (mg/mL)	1.87 (2.25)	1.94 (2.63)	2.06 (2.50)	0.718	1.94 (2.47)	1.84 (2.60)	2.23 (2.80)	0.082	2.00 (2.82)	1.90 (2.43)	1.98 (2.41)	0.897

^§^*p* for a test for inter-quintile trend. ^#^ E%/d, percentage of energy intake per day. * Plasma metabolic and cardiovascular biomarkers were measured after overnight fasting in a subset of 2402 study participants randomly sampled from the four centers (Varese, Turin, Naples, and Ragusa).

**Table 2:** Risk of colon and rectal cancers associated with replacement of 3% of energy from animal proteins with vegetable proteins.

	Colon Cancer	Rectal Cancer
All Participants	No. Cases = 438	No. Cases = 101
Animal proteins replaced with vegetable proteins		
HR ^1^ (95% CI)	1.11 (0.99–1.25)	0.76 (0.60–0.97)
HR ^2^ (95% CI)	1.12 (0.99–1.27)	0.71 (0.55–0.92)
Animal proteins replaced with vegetable proteins from high-GI foods		
HR ^1^ (95% CI)	1.23 (1.08–1.40)	0.71 (0.54–0.93)
HR ^2^ (95% CI)	1.23 (1.07–1.40)	0.68 (0.51–0.89)
Animal proteins replaced vegetable proteins from low-GI foods		
HR ^1^ (95% CI)	0.93 (0.79–1.09)	0.88 (0.64–1.20)
HR ^2^ (95% CI)	0.93 (0.79–1.11)	0.82 (0.58–1.15)

^1^ Stratified by center, age, and sex and adjusted for energy, alcohol, and total fat intakes. ^2^ Also adjusted for BMI, waist–hip ratio, smoking, education, physical activity, and fiber intake.

**Table 3:** Risk of colon and rectal cancers associated with replacement of 3% of energy from animal proteins and animal protein sources with plant proteins.

	Colon Cancer	Rectal Cancer
	No. Cases = 438	No. Cases = 101
Animal sources replaced with vegetable proteins		
Processed and red meat		
HR ^1^ (95% CI)	1.18 (1.03–1.34)	0.71 (0.55–0.92)
HR ^2^ (95% CI)	1.21 (1.07–1.39)	0.66 (0.50–0.87)
Poultry		
HR ^1^ (95% CI)	1.08 (0.92–1.25)	0.72 (0.53–0.98)
HR ^2^ (95% CI)	1.07 (0.92–1.25)	0.70 (0.50–0.96)
Fish		
HR ^1^ (95% CI)	1.05 (0.90–1.24)	0.83 (0.58–1.17)
HR ^2^ (95% CI)	1.06 (0.90–1.24)	0.79 (0.55–1.13)
Eggs and dairy products		
HR ^1^ (95% CI)	1.09 (0.96–1.23)	0.80 (0.62–1.03)
HR ^2^ (95% CI)	1.11 (0.97–1.26)	0.74 (0.57–0.97)
Animal sources replaced with vegetable proteins from high-GI foods		
Processed and red meat		
HR ^1^ (95% CI)	1.31 (1.13–1.50)	0.67 (0.50–0.89)
HR ^2^ (95% CI)	1.32 (1.14–1.52)	0.63 (0.47–0.85)
Poultry		
HR ^1^ (95% CI)	1.17 (1.00–1.38)	0.69 (0.50–0.95)
HR ^2^ (95% CI)	1.15 (0.99–1.36)	0.67 (0.48–0.94)
Fish		
HR ^1^ (95% CI)	1.15 (0.97–1.36)	0.79 (0.541.13)
HR ^2^ (95% CI)	1.15 (0.97–1.36)	0.76 (0.52–1.10)
Eggs and dairy products		
HR ^1^ (95% CI)	1.22 (1.06–1.39)	0.74 (0.56–0.99)
HR ^2^ (95% CI)	1.22 (1.06–1.40)	0.70 (0.52–0.94)
Animal sources replaced with vegetable proteins from low-GI foods		
Processed and red meat		
HR ^1^ (95% CI)	0.98 (0.82–1.16)	0.82 (0.58–1.14)
HR ^2^ (95% CI)	1.00 (0.83–1.19)	0.75 (0.52–1.08)
Poultry		
HR ^1^ (95% CI)	0.88 (0.72–1.07)	0.84 (0.57–1.23)
HR ^2^ (95% CI)	0.87 (0.72–1.07)	0.80 (0.53–1.21)
Fish		
HR ^1^ (95% CI)	0.86 (0.70–1.05)	0.96 (0.63–1.44)
HR ^2^ (95% CI)	0.87 (0.70–1.06)	0.90 (0.59–1.39)
*Eggs and dairy products*		
HR ^1^ (95% CI)	0.91 (0.77–1.07)	0.91 (0.66–1.24)
HR ^2^ (95% CI)	0.92 (0.78–1.10)	0.83 (0.59–1.19)

^1^ Stratified by center, age, and sex and adjusted for energy, alcohol, and total fat intakes. ^2^ Also adjusted for BMI, waist–hip ratio, smoking, education, physical activity, and fiber intake.

## Data Availability

Raw data cannot be made freely available because of restrictions imposed by the Ethical Committee that do not allow open/public sharing of data of individuals. However, aggregated data are available for other researchers upon request. Requests should be sent to Dr. Sabina Sieri (sabina.sieri@istitutotumori.mi.it).

## Supplementary Materials

The following supporting information can be downloaded at: https://www.mdpi.com/article/10.3390/cancers14122917/s1: Table S1. Risks of colon and rectal cancer associated with replacement of 3% of energy from animal protein with vegetable protein, stratified by body mass index (≤25 and >25). Table S2. Risk of colon and rectal cancer associated with replacement of 3% of energy from animal protein and animal protein sources with plant protein, stratified by body mass index (≤25 and >25).
