# Supplementary material for: Dietary Intakes of Animal and Plant Proteins and Risk of Colorectal Cancer: The EPIC-Italy Cohort
Source: Cancers (Basel). 2022 Jun 13;14(12):2917. doi: 10.3390/cancers14122917 (PMC9221300; doi:10.3390/cancers14122917)
Supplement: Supplementary file 1 [file cancers-14-02917-s001.zip › cancers-1684605-supplementary.pdf]

**Table S1. Risks of colon and rectal cancer associated with replacement of 3% of energy from animal protein with vegetable protein, stratified by body mass index (≤ 25 and >25)**				
	**Colon cancer**	**P for interaction**	**Rectal cancer**	**P for interaction [3]**
Body mass index ≤25	**n. cases=157**		**n. cases =39**	
**Animal protein replaced with vegetable protein**				
HR[1] (95% CI)	1.21 (0.98-1.48)		0.68 (0.45-1.01)	
HR[2] (95% CI)	**1.28 (1.03-1.59)**		**0.58 (0.38-0.89**	
**Animal protein replaced with vegetables protein from high GI foods**				
HR[1] (95% CI)	**1.41 (1.13-1.77)**		**0.60 (0.38-0.95)**	
HR[2] (95% CI)	**1.45 (1.15-1.84)**		**0.55 (0.34-0.88)**	
**Animal protein replaced with vegetable protein from low GI foods**				
HR[1] (95% CI)	0.91 (0.69-1.21)		0.83 (0.50-1.37)	
HR[2] (95% CI)	0.97 (0.72-1.31)		0.68 (0.38-1.21)	
Body mass index >25	**n. cases=281**		**n. cases =62**	
**Animal protein replaced with vegetable protein**				
HR[1] (95% CI)	1.07 (0.92-1.24)	0.898	0.83 (0.61-1.12)	0.294
HR[2] (95% CI)	1.07 (0.92-1.24)	0.836	0.81 (0.58-1.13)	0.551
**Animal protein replaced with vegetables protein from high GI foods**				
HR[1] (95% CI)	1.15 (0.98-1.35)	0.499	0.79 (0.56-1.12)	0.764
HR[2] (95% CI)	1.14 (0.97-1.34)	0.540	0.78 (0.54-1.12)	0.870
**Animal protein replaced with vegetable protein from low GI foods**				
HR[1] (95% CI)	0.95 (0.78-1.15)	0.153	0.92 (0.62-1.37)	0.265
HR[2] (95% CI)	0.93 (0.75-1.15)	0.185	0.91 (0.59-1.40)	0.435

[1] Stratified by center, age, sex and adjusted for energy, alcohol and total fat intake. [2] Additionally adjusted for BMI, waist-hip ratio, smoking, education, physical activity and fiber intake. [3] Test for heterogeneity comparing BMI ≤25 and BMI >25.

Table S2. Risk of colon and rectal cancer associated with replacement of 3% of energy from animal protein and animal protein sources with plant protein, stratified by body mass index (≤ 25 and >25)				
	**Colon cancer**	**P for interaction**	**Rectal cancer**	**P for interaction** [3]
Body mass index ≤25	**n. cases=157**		**n. cases =39**	
**Animal sources replaced with vegetable protein**				
Processed and red meat				
HR^1 (95% CI)	1.15 (0.92-1.44)		**0.62 (0.41-0.95)**	
HR^2 (95% CI)	1.23 (0.97-1.56)		**0.53 (0.34-0.83)**	
Poultry				
HR^1 (95% CI)	1.17 (0.90-1.53)		0.67 (0.40-1.12)	
HR^2 (95% CI)	1.20 (0.92-1.58)		0.62 (0.36-1.07)	
Fish				
HR^1 (95% CI)	1.25 (0.92-1.68)		0.74 (0.42-1.31)	
HR^2 (95% CI)	1.26 (0.93-1.71)		0.70 (0.39-1.25)	
Egg and dairy products				
HR^1 (95% CI)	**1.24 (1.01-1.53)**		0.72 (0.48-1.10)	
HR^2 (95% CI)	**1.32 (1.05-1.66)**		**0.61 (0.40-0.96)**	
**Animal sources replaced with vegetable protein from high GI foods**				
Processed and red meat				
HR^1 (95% CI)	**1.35 (1.06-1.72)**		**0.56 (0.35-0.90)**	
HR^2 (95% CI)	**1.40 (1.09-1.80)**		**0.50 (0.31-0.82)**	
Poultry				
HR^1 (95% CI)	**1.33 (1.01-1.75)**		0.61 (0.36-1.06)	
HR^2 (95% CI)	**1.35 (1.02-1.79)**		0.59 (0.33-1.05)	
Fish				
HR^1 (95% CI)	**1.43 (1.05-1.95)**		0.68 (0.37-1.23)	
HR^2 (95% CI)	**1.44 (1.05-1.96)**		0.66 (0.36-1.23)	
Eggs and dairy products				
HR^1 (95% CI)	**1.48 (1.17-1.87)**		0.64 (0.39-1.04)	
HR^2 (95% CI)	**1.53 (1.20-1.97)**		**0.58 (0.35-0.95)**	
**Animal sources replaced with vegetable protein from low GI foods**				
Processed and red meat				
HR^1 (95% CI)	0.85 (0.63-1.15)		0.77 (0.45-1.31)	
HR^2 (95% CI)	0.91 (0.66-1.25)		0.62 (0.34-1.13)	
Poultry				
HR^1 (95% CI)	0.84 (0.60-1.18)		0.84 (0.45-1.58)	
HR^2 (95% CI)	0.88 (0.62-1.25)		0.72 (0.36-1.44)	
Fish				
HR^1 (95% CI)	0.90 (0.63-1.30)		0.93 (0.47-1.82)	
HR^2 (95% CI)	0.93 (0.64-1.35)		0.81 (0.40-1.66)	
Eggs and dairy products				

HR[1] (95% CI)	0.93 (0.70-1.24)		0.87 (0.52-1.47)	
HR[2] (95% CI)	1.00 (0.73-1.35)		0.70 (0.39-1.28)	
Body mass index>25	**n. cases=281**		**n. cases =62**	
**Animal sources replaced with vegetable protein**				
Processed and red meat				
HR[1] (95% CI)	**1.21 (1.03-1.43)**	0.063	0.79 (0.57-1.10)	0.464
HR[2] (95% CI)	**1.22 (1.03-1.45)**	0.072	0.78 (0.54-1.11)	0.411
Poultry				
HR[1] (95% CI)	1.04 (0.87-1.25)	0.720	0.78 (0.53-1.14)	0.754
HR[2] (95% CI)	1.02 (0.85-1.23)	0.595	0.78 (0.52-1.17)	0.776
Fish				
HR[1] (95% CI)	0.99 (0.82-1.21)	0.376	0.90 (0.58-1.41)	0.650
HR[2] (95% CI)	0.98 (0.81-1.20)	0.328	0.88 (0.55-1.41)	0.627
Egg and dairy products				
HR[1] (95% CI)	1.02 (0.88-1.19)	0.195	0.85 (0.62-1.18)	0.927
HR[2] (95% CI)	1.03 (0.88-1.20)	0.243	0.83 (0.59-1.18)	0.661
**Animal sources replaced with vegetable protein from high GI foods**				
Processed and red meat				
HR[1] (95% CI)	**1.30 (1.09-1.55)**	0.066	0.76 (0.53-1.10)	0.286
HR[2] (95% CI)	**1.30 (1.09-1.56)**	0.073	0.75 (0.51-1.10)	0.398
Poultry				
HR[1] (95% CI)	1.11 (0.91-1.34)	0.732	0.75 (0.50-1.13)	0.757
HR[2] (95% CI)	1.08 (0.89-1.31)	0.607	0.76 (0.49-1.16)	0.768
Fish				
HR[1] (95% CI)	1.06 (0.86-1.30)	0.412	0.88 (0.55-1.40)	0.675
HR[2] (95% CI)	1.04 (0.85-1.28)	0.356	0.85 (0.52-1.39)	0.640
Eggs and dairy products				
HR[1] (95% CI)	1.11 (0.94-1.31)	0.190	0.81 (0.56-1.18)	0.932
HR[2] (95% CI)	1.10 (0.93-1.31)	0.236	0.80 (0.54-1.17)	0.663
**Animal sources replaced with vegetable protein from low GI foods**				
Processed and red meat				
HR[1] (95% CI)	1.06 (0.86-1.32)	0.060	0.88 (0.58-1.33)	0.303
HR[2] (95% CI)	1.07 (0.85-1.34)	0.069	0.88 (0.53-1.46)	0.426
Poultry				
HR[1] (95% CI)	0.91 (0.71-1.14)	0.759	0.87 (0.54-1.40)	0.781
HR[2] (95% CI)	0.89 (0.69-1.13)	0.629	1.14 (0.69-1.90)	0.809
Fish				
HR[1] (95% CI)	0.86 (0.68-1.10)	0.394	1.01 (0.60-1.68)	0.680
HR[2] (95% CI)	0.86 (0.67-1.10)	0.342	0.99 (0.57-1.70)	0.649
Eggs and dairy products				
HR[1] (95% CI)	0.91 (0.74-1.10)	0.189	0.94 (0.63-1.39)	0.932
HR[2] (95% CI)	0.90 (0.73-1.12)	0.234	0.92 (0.60-1.42)	0.655

[1] Stratified by center, age, sex and adjusted for energy, alcohol and total fat intake. [2] Additionally adjusted also for BMI, waist-hip ratio, smoking, education, physical activity and fiber intake. [3] Test for heterogeneity comparing BMI ≤25 and BMI >25.
